# Supplementary figures and images for: Disruption of PIKFYVE causes congenital cataract in human and zebrafish
Source: eLife. 2022 Jan 13;11:e71256. doi: 10.7554/eLife.71256 (PMC8758139; doi:10.7554/eLife.71256)

Source data for the full raw unedited blots in Figure 2D.

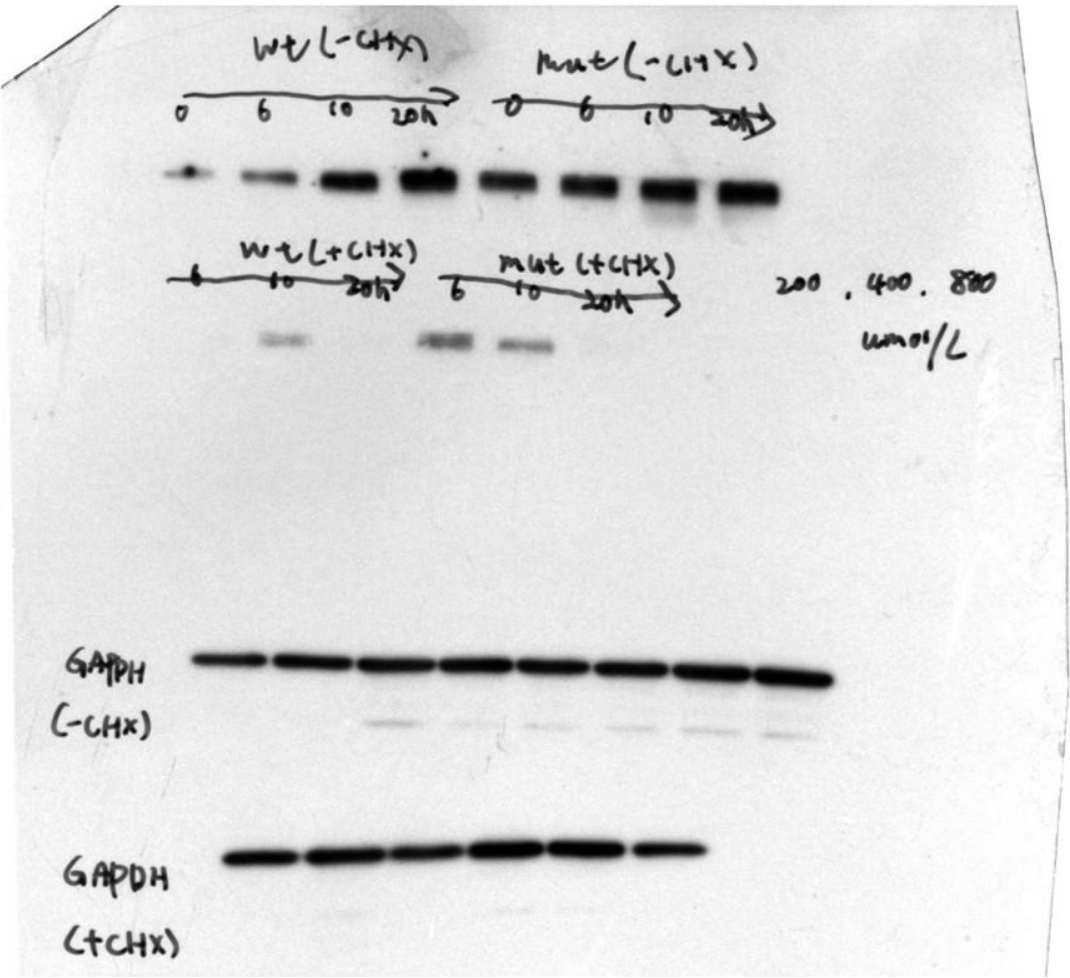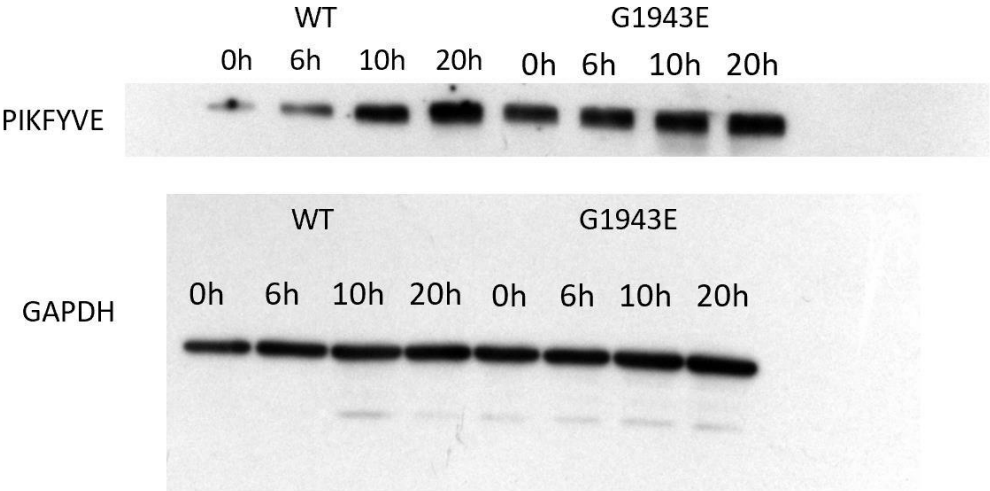

Supplement: Figure 2—source data 2. [file elife-71256-fig2-data2.pdf]
